# A simple method suitable to study *de novo* root organogenesis

**DOI:** 10.3389/fpls.2014.00208

**Published:** 2014-05-15

**Authors:** Xiaodong Chen, Yuliang Qu, Lihong Sheng, Jingchun Liu, Hai Huang, Lin Xu

**Affiliations:** ^1^National Laboratory of Plant Molecular Genetics, Institute of Plant Physiology and Ecology, Shanghai Institutes for Biological Sciences – Chinese Academy of SciencesShanghai, China; ^2^Shanghai Center for Plant Stress Biology – Chinese Academy of SciencesShanghai, China; ^3^College of Life and Environment Sciences, Shanghai Normal UniversityShanghai, China

**Keywords:** *de novo* root organogenesis, B5 medium, leaf age, sucrose, adventitious root, plant regeneration

## Abstract

*De novo* root organogenesis is the process in which adventitious roots regenerate from detached or wounded plant tissues or organs. In tissue culture, appropriate types and concentrations of plant hormones in the medium are critical for inducing adventitious roots. However, in natural conditions, regeneration from detached organs is likely to rely on endogenous hormones. To investigate the actions of endogenous hormones and the molecular mechanisms guiding *de novo* root organogenesis, we developed a simple method to imitate natural conditions for adventitious root formation by culturing *Arabidopsis thaliana* leaf explants on B5 medium without additive hormones. Here we show that the ability of the leaf explants to regenerate roots depends on the age of the leaf and on certain nutrients in the medium. Based on these observations, we provide examples of how this method can be used in different situations, and how it can be optimized. This simple method could be used to investigate the effects of various physiological and molecular changes on the regeneration of adventitious roots. It is also useful for tracing cell lineage during the regeneration process by differential interference contrast observation of β-glucuronidase staining, and by live imaging of proteins labeled with fluorescent tags.

## INTRODUCTION

Unlike animals, plants cannot move independently. Therefore, plants have evolved various kinds of abilities to survive under severe environmental conditions. *De novo* organogenesis, in which adventitious shoots and roots regenerate from detached or wounded tissues or organs, is one of these survival strategies (Duclercq et al., 2011; Sugimoto et al., 2011; Xu and Huang, 2014). *De novo* shoot and root organogenesis can be induced both in tissue culture and under natural conditions. In tissue culture conditions, detached plant tissues or organs are usually cultured on nutrient-enriched media containing appropriate plant hormones such as auxin and cytokinin, and rooting or shooting from explants can be manipulated (Skoog and Miller, 1957). Under natural conditions, detached organs can generate adventitious shoots and roots to ultimately form a whole plant under certain circumstances. In this situation, organogenesis relies on endogenous hormones (Birnbaum and Sanchez Alvarado, 2008; Xu and Huang, 2014).

When detached, many types of plant organs initiate *de novo* root organogenesis in nature. The newly formed adventitious roots can ensure the water supply for the subsequent whole plant regeneration (De Klerk et al., 1999; Xu and Huang, 2014). During this process, adventitious roots primarily initiate from the procambium or cambium cells (Greenwood et al., 2001; Ahkami et al., 2009; Correa Lda et al., 2012; Ahkami et al., 2013; Liu et al., 2014), which are the adult stem cells located in the vascular tissues of aerial organs. Thus, these stem cells may have the potential to initiate plant *de novo* organogenesis. In addition, auxin is known to be essentially required for regeneration of adventitious roots (De Klerk et al., 1999; Greenwood et al., 2001; Pop et al., 2011; Correa Lda et al., 2012; da Costa et al., 2013; Verstraeten et al., 2013; Liu et al., 2014).

To study the underlying mechanism guiding *de novo* organogenesis, we developed a simple method mimicking natural conditions to regenerate adventitious roots from leaf explants of the model plant *Arabidopsis thaliana* (Liu et al., 2014). We revealed two steps of cell fate transition in *de novo* root organogenesis using this method. Accumulation of high levels of auxin in the vasculature of leaf explants induced the expression of *WUSCHEL-RELATED HOMEOBOX 11* (*WOX11*). This triggered the first-step cell fate transition from procambium or its nearby parenchyma cells to root founder cells. The cell division occurred during the second-step cell fate transition from root founder cells to root primordium cells, which expressed the root quiescent center (QC) marker *WOX5* (Liu et al., 2014).

The major difference of the method described here from other tissue culture methods reported previously is that no additional hormones are present in the medium. Therefore, the regeneration of adventitious roots from explants depends only on endogenous hormones. We propose that this method will be useful to study *de novo* root organogenesis at the physiological, cellular, and molecular levels. Here, we describe in detail the use of this method and its applications, in combination with different microscopic techniques including live imaging. Also, we describe how to improve the efficiency of this method by optimizing factors such as leaf age and nutrients in the medium.

## RESULTS

### REGENERATION OF ADVENTITIOUS ROOTS FROM LEAF EXPLANTS ON HORMONE-FREE MEDIUM

**FIGURE 1:**
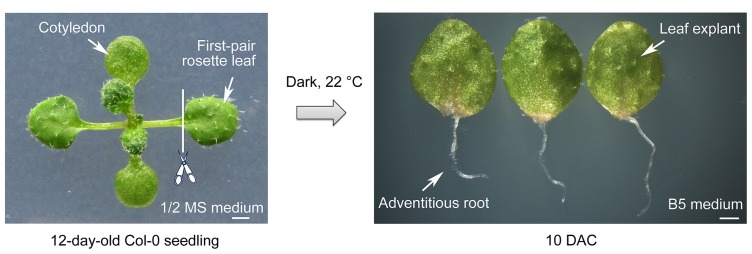
**Method for *de novo* root organogenesis**. Scale bar: 1 mm.

To study the mechanisms underlying *de novo* root organogenesis, we established a method using *Arabidopsis* leaf explants cultured on B5 medium (Liu et al., 2014). Seeds of *Arabidopsis* were grown on 1/2 Murashige and Skoog (MS) medium. The 12-day-old seedlings had developing first-pair rosette leaves with emerging third and fourth ones (**Figure 1**). The first-pair rosette leaves were cut at the position between petiole and blade, and the blade part was then cultured in the dark on B5 medium, which is free of additive plant hormones. Adventitious roots emerging from the mid-vein near the wound could be observed by 6 days after culture (DAC). The whole process was easy to perform and the results were reliable. In our culture conditions, almost all leaf explants had generated adventitious roots at 10 DAC (**Figure 1**).

### EFFECTS OF LEAF AGE ON REGENERATION ABILITY

**FIGURE 2:**
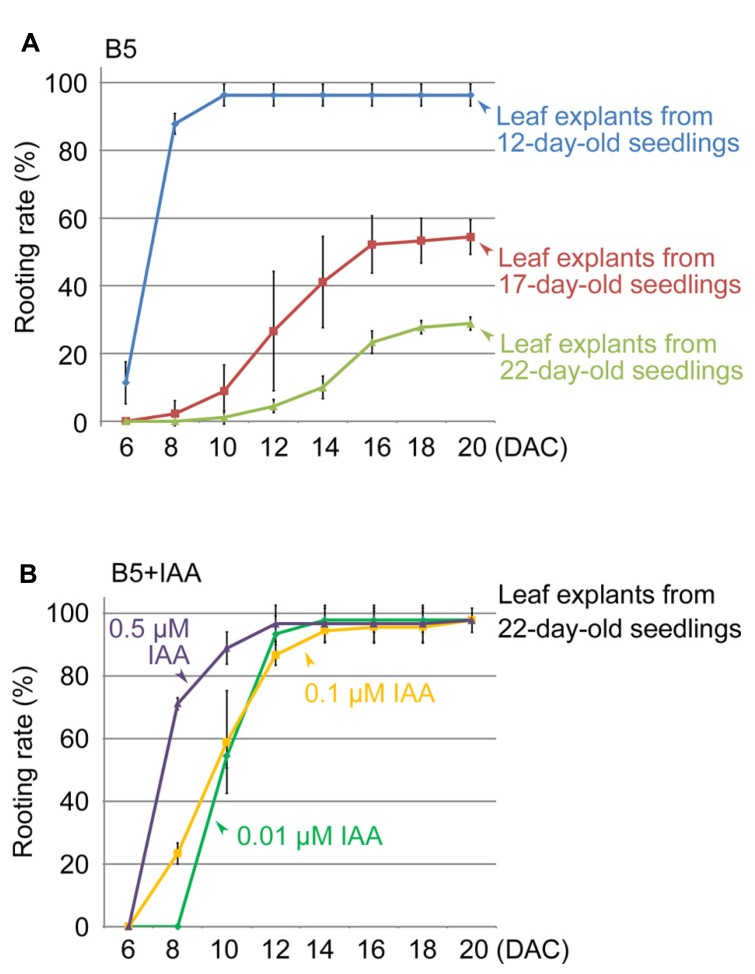
**Effects of leaf age and auxin concentration on rooting rate of leaf explants. (A)** Rooting rate of leaf explants from 12-, 17-, and 22-day-old seedlings. **(B)** Effect of IAA in B5 medium to rescue rooting defect of leaf explants from 22-day-old seedlings. Bars show s.d. with three biological repeats. *N* = 30 in each individual repeat.

Next, we used the method to study different factors that affect regeneration of adventitious roots. We first tested leaves of different ages, comparing the first-pair rosette leaves from 17- and 22-day-old seedlings with those from 12-day-old seedlings. Our results showed that the regeneration rate decreased with increasing age of the first-pair rosette leaves (**Figure 2A**). Leaf explants from 22-day-old seedlings showed only a 29% rooting rate at 20 DAC (**Figure 2A**), suggesting that the age of leaf explants is an important factor affecting adventitious root formation.

In this method, endogenous auxin plays a primary role in initiating adventitious root formation from leaf explants (Liu et al., 2014). Therefore, we tested whether the reduced regeneration ability of older leaves is because of a lack of free auxin. We cultured the first-pair rosette leaf explants from 22-day-old seedlings on B5 medium containing indole-3-acetic acid (IAA). Our data showed that 0.01 μM IAA could partly rescue the regeneration defect caused by the increased ages of leaves, and higher concentrations of auxin in B5 medium (0.1 and 0.5 μM) had an even stronger rescuing effect on rooting (**Figure 2B**). These results suggested that the loss of regeneration ability in older leaf explants is at least partly because of low levels of endogenous free auxin.

### CARBOHYDRATE IS REQUIRED FOR REGENERATION OF ADVENTITIOUS ROOTS

**FIGURE 3:**
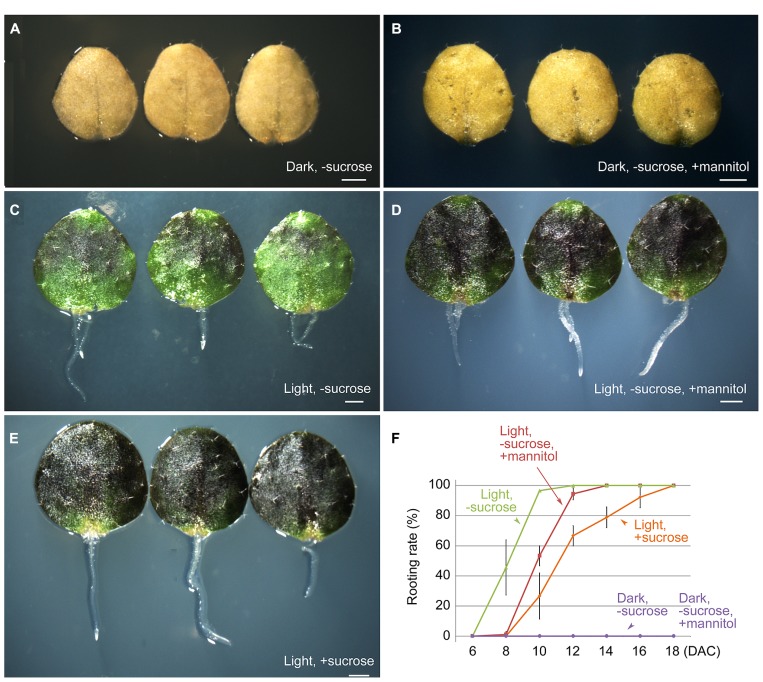
**Effects of light and sucrose on adventitious root formation from leaf explants. (A,B)** Leaf explants at 10 DAC in the dark on B5 medium without sucrose **(A)** or with 100 mM mannitol instead of sucrose **(B)** showing no regeneration of adventitious roots. **(C,D)** Leaf explants at 10 DAC under light conditions on B5 medium without sucrose **(C)** or with 100 mM mannitol instead of sucrose **(D)**. **(E)** Leaf explants at 10 DAC under light conditions on B5 medium with sucrose. **(F)** Rooting rate analyses of cultured leaf explants for **(A–E)**. Leaf explants were cut from 12-day-old seedlings. Bars in **(F)** show SD with three biological repeat. *N* = 30 for each repeat. Scale bars: 1 mm in **(A–E)**.

Next, we tested the effect of carbohydrate supplementation on the ability of the first-pair rosette leaf explants to form adventitious roots. The B5 medium used for culturing leaf explants contains 100 mM sucrose as the energy source. When leaf explants were cultured in the dark on B5 medium without sucrose or with 100 mM mannitol instead of sucrose, regeneration of adventitious roots was completely blocked (**Figures 3A,B,F**). This result suggested that a carbohydrate that can be used by plants to produce energy is necessary for regeneration. However, under light conditions, leaf explants were able to root normally on B5 medium without sucrose or with mannitol instead of sucrose (**Figures 3C,D,F**), similar to those cultured on B5 medium with sucrose (**Figures 3E,F**). This finding suggested that photosynthesis of leaf explants can supply sufficient energy for rooting. Sugar functions not only as a nutrient, but also as a signal for plant development and in various physiological processes (Rolland et al., 2006). Recently, it was reported that carbohydrates may crosstalk with auxin in adventitious root formation (Ahkami et al., 2013). In future research, it will be interesting to test whether sucrose functions as a signal molecule in the regeneration process.

### TRACING CELL LINEAGE USING MICROSCOPY TECHNIQUES

**FIGURE 4:**
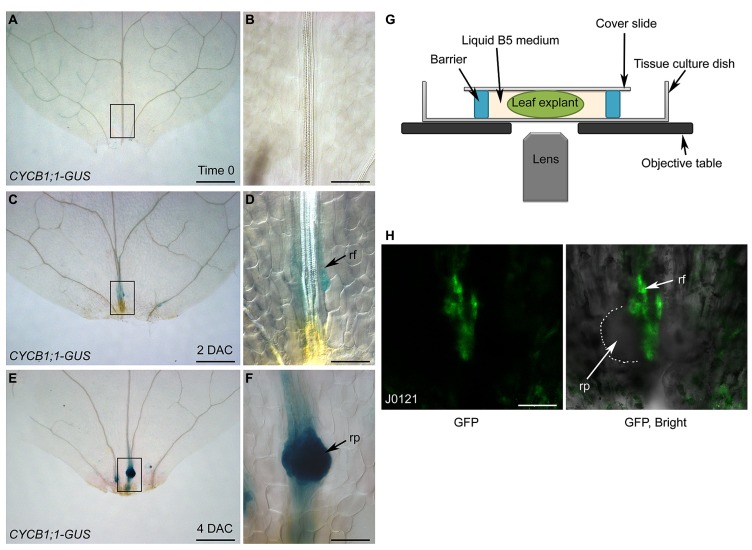
**Analysis of cell lineage by DIC and confocal microscopies. (A–F)** GUS staining of time-0 **(A,B)**, 2-DAC **(C,D)**, and 4-DAC **(E,F)** leaf explants from *CYCB1;1-GUS* plants, cultured on B5 medium. **(B,D,F)** show magnifications of boxed regions in **(A,C,E)**, respectively. Leaf explants were cut from 12-day-old seedlings. **(G)** Diagram of system for confocal observation of *de novo* root regeneration from leaf explants. **(H)** GFP fluorescence of 4-DAC leaf explants from J0121 plants. rf, root founder cell; rp, root primordium. Scale bars: 500 μm in **(A,C,E)**; 100 μm in **(B,D,F,H)**.

Using differential interference contrast (DIC) microscopy, cell lineage can be analyzed by tracing key genes labeled with β*-glucuronidase* (GUS). Here, we used *CYCB1;1-GUS* (Colon-Carmona et al., 1999), which marks dividing cells, as an example. The leaf explant prior to culturing (i.e., time 0) showed faint GUS staining (**Figures 4A,B**), suggesting that cells in the leaf explants were not in a rapidly dividing state at this stage. In 2-DAC leaf explants, there was weak GUS staining in the root founder cells that were about to divide (**Figures 4C,D**). In 4-DAC leaf explants, there was clear and strong GUS staining in the newly formed root primordium, showing that cells in the root primordium are dividing rapidly at this stage (**Figures 4E,F**).

Although GUS staining together with DIC observation can be used to analyze the cell lineage, the disadvantage is that the fixing and staining processes prevent successive observations of *de novo* root regeneration. This obstacle can be overcome by using live imaging techniques. For example, when green fluorescent protein (GFP) was used to label key factors, GFP fluorescence could be observed by confocal microscopy. Leaf explants were cultured in liquid B5 medium between the cover slide and the tissue culture dish (**Figure 4G**). Using a confocal system, the cell lineage can be traced over time by observing the same leaf explant continuously. Here, we show confocal images of a leaf explant carrying the J0121 GFP marker, which labels the pericycle-like adult stem cells that will undergo regeneration (Laplaze et al., 2005; Sugimoto et al., 2010), as an example (**Figure 4H**).

### EXTENDING OTHER APPLICATIONS OF THE ROOTING METHOD

**FIGURE 5:**
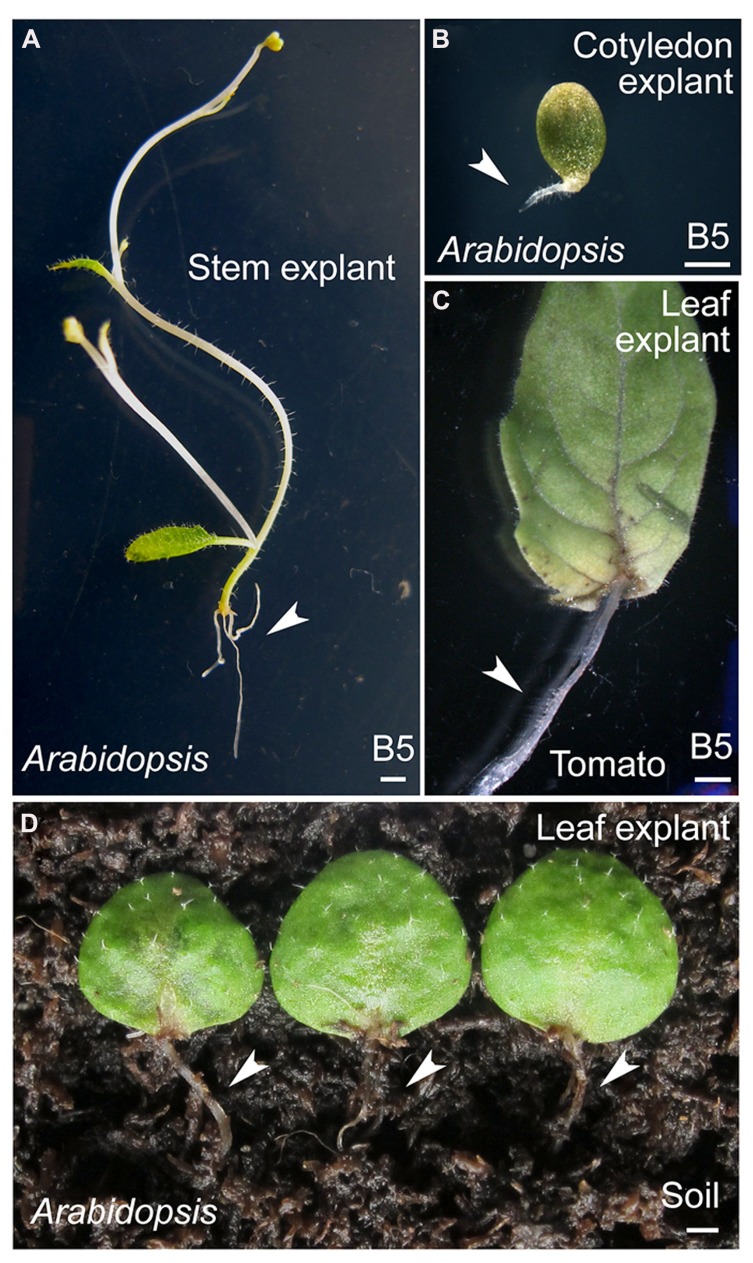
**Regeneration of adventitious roots from *Arabidopsis* organs and tomato leaves. (A,B)** Regeneration of adventitious roots from stem **(A)** or cotyledon **(B)** explants of 20-day-old or 7-day-old *Arabidopsis* seedlings, respectively. **(C)** Regeneration of adventitious roots from tomato leaflet explants. Leaflet was detached from the first pair of compound leaves of 15-day-old seedlings. **(D)** Regeneration of adventitious roots from leaf explants of 15-day-old *Arabidopsis* seedlings on surface of wet soil. Of 90 leaf explants tested, 48 had regenerated adventitious roots at 15 DAC. Arrowheads in **(A–D)** indicate adventitious roots regenerated from explants. Scale bars: 1 mm in **(A–D)**.

In addition to leaf explants, explants from other aerial organs of *Arabidopsis*, such as cotyledons and stems, have been used as materials in this method (**Figures 5A,B**). Leaf explants from other dicot model plants, such as tomato (**Figure 5C**), are also able to regenerate adventitious roots. Furthermore, *Arabidopsis* leaf explants can be cultured on the surface of wet soil to regenerate adventitious roots (**Figure 5D**). Thus, this method may be widely used to study *de novo* root organogenesis.

## DISCUSSION

Most previous studies on adventitious root formation from detached aerial organs have used exogenous auxin in the medium to induce rooting (De Klerk et al., 1999; Correa Lda et al., 2012; Verstraeten et al., 2013; Welander et al., 2014). In these conditions, explants show a strong regeneration ability. However, the use of exogenous hormones may result in bypassing the actions of endogenous hormones, and/or affect upstream events such as regulatory events that control hormone levels after wounding. The method described in this study avoids use of additive plant hormones, and differs from most methods reported previously. Thus, the method creates an environment that imitates natural conditions. This method allowed us to analyze hormone actions at early stages of regeneration, including the molecular mechanisms guiding free auxin production, auxin polar transport, and the proposed wound signal that acts upstream of auxin action (Liu et al., 2014).

In this study, we show that leaf age is critical for the regeneration of adventitious roots from leaf explants. The decreased regeneration ability of older leaves is probably a result of insufficient free auxin level in their tissues. Consistent with this idea, a previous study showed that the auxin levels declined with leaf age (Shoji et al., 1951). However, we cannot exclude other possibilities. For example, the polar transport of auxin may not occur as efficiently in older leaves. Auxin is of primary importance in *de novo* root organogenesis (Liu et al., 2014). Therefore, in this method, it is important to use young leaves with higher endogenous auxin levels for efficient regeneration of adventitious roots. Age may also be a factor when other aerial organs are used as explants for rooting.

The process of regeneration from explants may require considerable energy, since it includes not only stem cell fate transition but also rapid cell division to form the root primordium. Therefore, a carbohydrate energy source, such as sucrose, must be included in the medium when explants are cultured in darkness. For green leaf explants that are cultured in light conditions, photosynthesis may produce enough energy to fuel the regeneration process. In addition, leaf explants can be cultured in liquid B5 medium with sucrose, making it possible to use live imaging techniques to observe *de novo* root organogenesis over time by confocal microscopy.

In natural conditions, detached leaves of some species, such as those in the Crassulaceae family, readily regenerate adventitious roots on the soil. One possible reason for this is their ability to retain water and nutrients after they are detached. We tested *Arabidopsis* and tomato, and found that leaf explants of both plant species were able to regenerate adventitious roots. This suggested that the regeneration of adventitious roots from detached leaves might be an ability conserved among dicots.

## MATERIALS AND METHODS

### PLANT MATERIALS AND CULTURE CONDITIONS

*Arabidopsis thaliana* (Col-0) and tomato (*Solanum lycopersicum*) were used in this study. Seeds were treated with 75% alcohol for 20 min and then washed four times with sterilized water, each for 20 min. The seeds were kept at 4°C for 2 days and then placed on 1/2 MS medium (half-strength of MS basal medium with 1% sucrose, 1% agar, and 0.5 g/L MES, pH5.7; Murashige and Skoog, 1962). *Arabidopsis* and tomato were grown at 22 and 25°C, respectively, with a 16-h light (~5000 Lux, cool white fluorescent lamp) and 8-h dark photoperiod in a plant tissue culture chamber (Percival, Perry, GA, USA). Explants were cultured on B5 medium (Gamborg B5 basal medium with 0.5 g/L MES, 100 mM sucrose, and 0.8% agar, pH 5.7; Gamborg et al., 1968) or B5 medium without sucrose, under light conditions described above or in darkness.

### GUS STAINING AND MICROSCOPY

For *CYCB1;1-GUS* staining, plant tissues were incubated in GUS assay solution (50 mM sodium phosphate buffer pH 7, 5 mM Na_2_EDTA, 2 mM K_3_Fe(CN)_6_, 2 mM K_4_Fe(CN)_6_, 0.1% Triton X-100, and 0.04% X-Gluc) at 37°C for 3 h. The stained tissues were incubated in 75% alcohol at 37°C for 12 h, and then in the chloral hydrate solution (200 g chloral hydrate; 20 g glycerol; 50 ml H_2_O; Tsuge et al., 1996) at 65°C for approximately 12 h, until tissues became transparent. The DIC observations were conducted using a Nikon Eclipse Ti microscope (Nikon, Tokyo, Japan). We used a ZEISS LSM510 META (Zeiss, Wetzlar, Germany) confocal microscopic system to observe GFP fluorescence.

## Conflict of Interest Statement

The authors declare that the research was conducted in the absence of any commercial or financial relationships that could be construed as a potential conflict of interest.
